# ARD-101, a gut-restricted TAS2R agonist, reduces hunger in adults and promotes weight loss in DIO mice with DPP-4 inhibition

**DOI:** 10.1016/j.molmet.2026.102340

**Published:** 2026-03-14

**Authors:** Zhenhuan Zheng, Jeremy H. Pettus, Alexa Warner, Bryan Jones, Megan Pugsley, Justin Stege, Brad Hirakawa, Manasi Jaiman, Jerlyn Tolentino, Tien-Li Lee, Timothy J. Kieffer

**Affiliations:** 1 Aardvark Therapeutics, 4370 La Jolla Village Drive, Suite 1050, San Diego, CA, 92122, USA; 2 Division of Endocrinology, Altman Clinical and Translational Research Institute (ACTRI), University of California, San Diego, 9452 Medical Center Drive L1W-515, La Jolla, CA, 92037, USA; 3 Life Sciences Institute, Department of Cellular and Physiological Sciences, Department of Surgery, School of Biomedical Engineering, University of British Columbia, Vancouver, BC, V6T1Z3, Canada

**Keywords:** TAS2R agonist, Denatonium acetate, Obesity, Hunger score, Diet-induced obese mice

## Abstract

**Objectives:**

Obesity management has limited oral pharmacotherapies. Bitter taste receptor (TAS2R) agonists may modulate hunger, satiety, and metabolism via gut-brain signaling. We evaluated denatonium acetate (DA), a gut-restricted TAS2R agonist, across preclinical and clinical settings, and explored its combination with sitagliptin (a dipeptidyl peptidase-4 [DPP-4] inhibitor).

**Methods:**

In mice transitioned to high-fat diet (HFD) or established with diet-induced obesity (DIO), we tested oral DA (20–80 mg/kg twice daily or 75 mg/kg once daily), a sitagliptin-formulated HFD, the combination, and subcutaneous tirzepatide, including a post-tirzepatide discontinuation phase, to assess weight trajectories and metabolic benefits. In randomized, placebo-controlled clinical studies, ARD-101 (oral DA) was evaluated in adults with obesity (200 mg twice daily for 28 days) and in healthy participants (single 800 mg).

**Results:**

In mice transitioned to HFD, DA reduced weight gain (up to 43.1%), decreased food intake, and improved glucose and lipid measures. In DIO mice, once-daily DA or sitagliptin-HFD prevented weight gain; the combination reduced body weight (−18.8%) with metabolic benefits. In a separate DIO mouse study, tirzepatide reduced weight by 23.7%. Following tirzepatide discontinuation, switching to DA plus sitagliptin-HFD limited weight regain comparable to continued tirzepatide. In adults with obesity, ARD-101 reduced weight versus placebo by 0.8 kg at Day 28 and 1.3 kg at end-of-study and decreased hunger and food cravings. It also altered gut hormone levels in healthy participants.

**Conclusions:**

Gut-restricted TAS2R agonism warrants further study for hyperphagia in Prader–Willi syndrome, and in combination with DPP-4 inhibition for obesity.

**Clinicaltrials.gov number:**

NCT05121441.

**Integrated research application system (IRAS) number:**

1011885.

## Highlights

• DA reduces weight gain, food intake, and improves metabolic markers in mice on HFD.
• DA and sitagliptin synergize to enhance weight loss and glucose control in DIO mice.
• Combination therapy sustains weight and metabolic benefits post-GLP-1RA cessation.
• Oral ARD-101 lowers hunger and supports metabolic health in adults with obesity.
• Oral ARD-101 alters gut hormone levels in healthy participants.

## 1 Introduction

Bitter taste receptors (TAS2Rs), a subset of G protein-coupled receptors, are traditionally known for their role in detecting bitter compounds in the oral cavity. However, growing evidence has revealed their widespread expression in extra-oral tissues, including the gastrointestinal tract, lungs, pancreas, and various immune and endocrine cells [1]. These extra-oral TAS2Rs are increasingly recognized for their diverse physiological roles, ranging from the modulation of hunger and metabolic processes to bronchodilation and anti-inflammatory effects [2,3]. In the gastrointestinal tract, TAS2Rs are expressed on enteroendocrine cells, where their activation by bitter compounds can stimulate the release of gut-derived peptide hormones involved in hunger regulation, satiation, and satiety [3]. This gut-brain signaling axis represents a promising therapeutic target for metabolic disorders, including obesity and hyperphagia-associated conditions like Prader–Willi syndrome (PWS) [4,5].

Clinical studies have shown that intragastric administration of bitter agents such as denatonium benzoate and quinine can acutely influence hunger ratings, gastrointestinal motility, and gut hormone secretion in humans [6,7]. ARD-101 is a novel oral formulation of denatonium acetate (DA), a TAS2R agonist, with a coating designed to prevent drug release in the oral cavity, thereby delivering DA directly to the gastrointestinal tract while avoiding activation of TAS2Rs in the oral cavity. This formulation strategy aims to leverage gut-restricted TAS2R signaling for metabolic benefit without triggering aversive taste responses. Moreover, DA shows minimal systemic absorption in mice (Aardvark Therapeutics, unpublished data) and humans [8], and appears to be well tolerated.

Activation of gastrointestinal TAS2Rs by bitter compounds such as denatonium benzoate has been shown to promote gut peptide release from enteroendocrine cells [9]; therefore, pairing DA with a dipeptidyl peptidase-4 (DPP-4) inhibitor such as sitagliptin provides a mechanistic strategy to prolong the half-life and exposure of incretin hormones (e.g., glucagon-like peptide-1 (GLP-1) and glucose-dependent insulinotropic polypeptide (GIP)) and other DPP-4-sensitive peptides. Sitagliptin has an established safety/tolerability profile and is generally weight-neutral as monotherapy [10,11]; combined with gut-restricted DA, this strategy was designed to sustain downstream incretin signaling and enhance satiety/metabolic effects while maintaining overall tolerability. Accordingly, in addition to evaluating DA across multiple dose levels in mice transitioned to a high-fat diet (HFD), we tested DA in combination with a sitagliptin-containing HFD in diet-induced obese (DIO) mice, including a post-tirzepatide discontinuation phase, to evaluate whether DPP-4 inhibition augments TAS2R-driven gut hormone signaling and translates into greater control of weight gain, food intake, and metabolic biomarkers. We also present a randomized, placebo-controlled Phase 2 proof-of-concept study of ARD-101 in adults living with obesity, assessing body weight, metabolic parameters, and Control of Eating Questionnaire (CoEQ) scores, as well as a randomized, placebo-controlled clinical pharmacology study in healthy adults, assessing the effects of ARD-101 on gut hormone secretion. Together, these preclinical and early clinical findings support DA as a gut-restricted TAS2R agonist with translational potential for treating chronic hunger and provide a mechanistic rationale for pairing DA with DPP-4 inhibition for the treatment of obesity.

## 2 Results

### 2.1 DA attenuates body weight gain and reduces daily food intake in mice on HFD with dose-responsive trends

**Figure 1:**
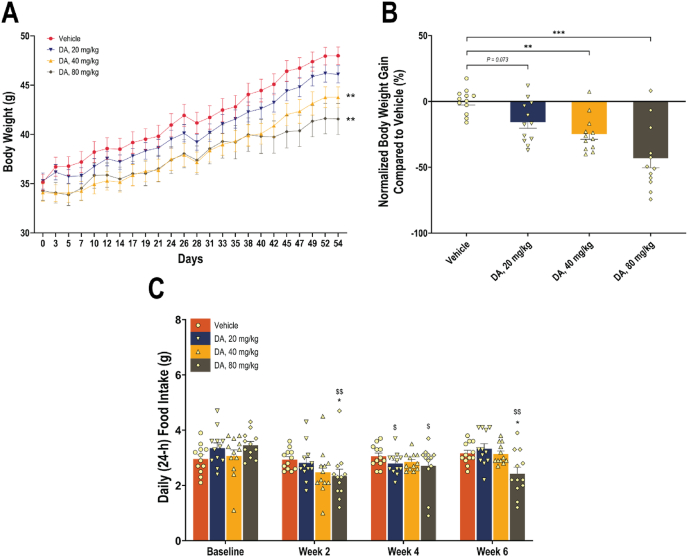
**Body weight changes and 24-h food intake in mice on HFD treated with DA or vehicle over eight weeks**. (A) Body weight trajectories (starting from individual housing) following twice-daily oral administration of DA at 20, 40, or 80 mg/kg, or vehicle. Data are presented as means (dot symbols) ± SEM (bars) for each treatment group (*N* = 12 per group). ∗∗*P* < 0.01, vs. the vehicle group at Day 54. (B) Percent body weight gain at study end, normalized to the vehicle group. Data are presented as means (bars) and SEM (error bars), with individual values overlaid (dots). ∗∗*P* < 0.01 and ∗∗∗*P* < 0.001, vs. the vehicle group. (C) 24-hour food intake at baseline and during treatment. Food consumption was recorded at baseline and at Weeks 2, 4, and 6. Data are presented as means (bars) and SEM (error bars) for all treatment groups (*N* = 12 per group), with individual values overlaid (dots). ∗*P* < 0.05, vs. the vehicle group at the corresponding time point. ^$^*P* < 0.05 and ^$$^*P* < 0.01, vs. baseline within the same treatment group.

In Study 1, over an eight-week treatment period in mice newly transitioned to ad libitum HFD, orally administered DA significantly attenuated body weight gain in a dose-dependent manner. As shown in Figure 1A, mice treated with DA at 20, 40, or 80 mg/kg twice daily exhibited reduced body weight trajectories compared to the vehicle-treated controls. Two-way ANOVA revealed a main effect of treatment on body weight gain (*F* (3, 44) = 2.845, *P* = 0.048). Final body weight gain, normalized to the vehicle group (Figure 1B), was reduced by 15.8%, 24.7%, and 43.1% in the low, medium, and high dose groups, respectively (*P* = 0.073, 0.0027, and <0.0001 vs. vehicle). These data support the potential of DA to limit weight gain in a preclinical model of obesity.

To evaluate the impact of orally administered DA on feeding behavior, daily food intake was measured at baseline and during Weeks 2, 4, and 6 of treatment. As shown in Figure 1C, DA treatment led to a reduction in 24-hour food intake across all dose levels compared to vehicle controls. Two-way ANOVA showed a trend toward significance for overall treatment effect (*F* (3, 44) = 2.644, *P* = 0.061) and a dose-dependent reduction in food intake was evident at Weeks 2 and 6.

### 2.2 DA significantly modulates key metabolic biomarkers in mice on HFD in a dose-dependent manner

**Figure 2:**
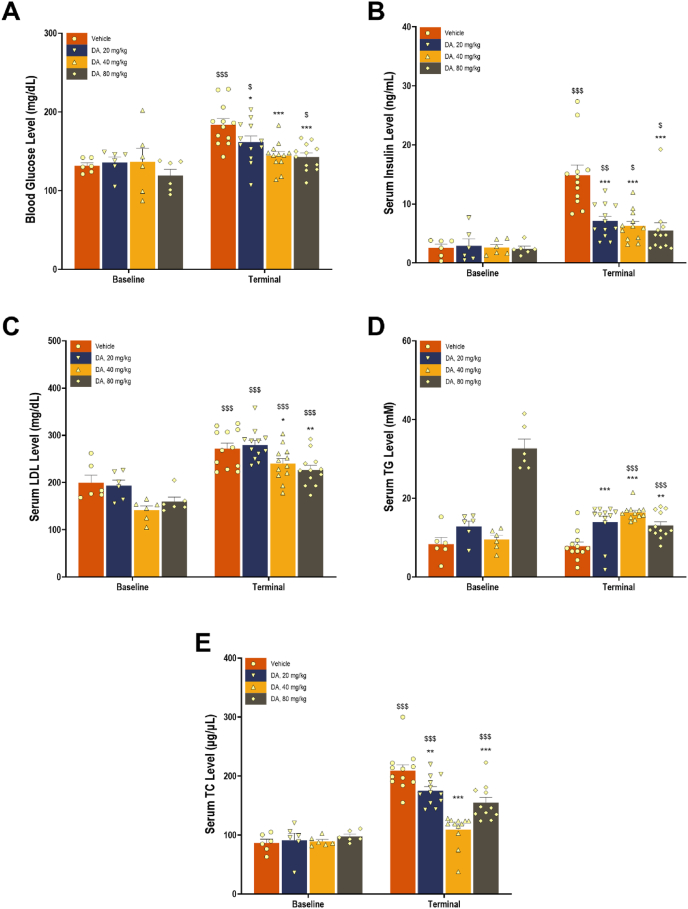
**Circulating metabolic biomarker levels in DIO mice treated with DA or vehicle**. Fasted levels of glucose (A), insulin (B), LDL (C), TG (D), and TC (E) were measured at baseline and after eight weeks of treatment. Data are presented as means (bars) and SEM (error bars), with individual values overlaid (dots). *N* = 6 per group at baseline; *N* = 12 per group at study end. ∗*P* < 0.05, ∗∗*P* < 0.01, and ∗∗∗*P* < 0.001, vs. the vehicle group at the same time point. ^$^*P* < 0.05, ^$$^*P* < 0.01, and ^$$$^*P* < 0.001 vs. baseline within the same treatment group.

To assess the metabolic effects of DA, five fasting circulating biomarkers, glucose, insulin, LDL, TG, and TC, were measured at baseline and at study termination. As shown in Figure 2A–E and summarized in Supplementary Table 1, oral administration of DA led to dose-dependent changes across all five metabolic biomarkers. For glucose, insulin, LDL, and TC (Figure 2A–C, and E), vehicle-treated mice showed rising levels over time, which were attenuated by DA in a dose-dependent fashion. TG levels (Figure 2D) were significantly reduced only at the highest dose (80 mg/kg), while minimal changes were observed at the lower doses. These findings indicate that DA exerts dose-responsive effects on glycemic and lipid parameters that extend beyond weight control.

### 2.3 Combined treatment with DA and sitagliptin induces body weight loss, reduces food intake, and improves glucose homeostasis and body composition in DIO mice

**Figure 3:**
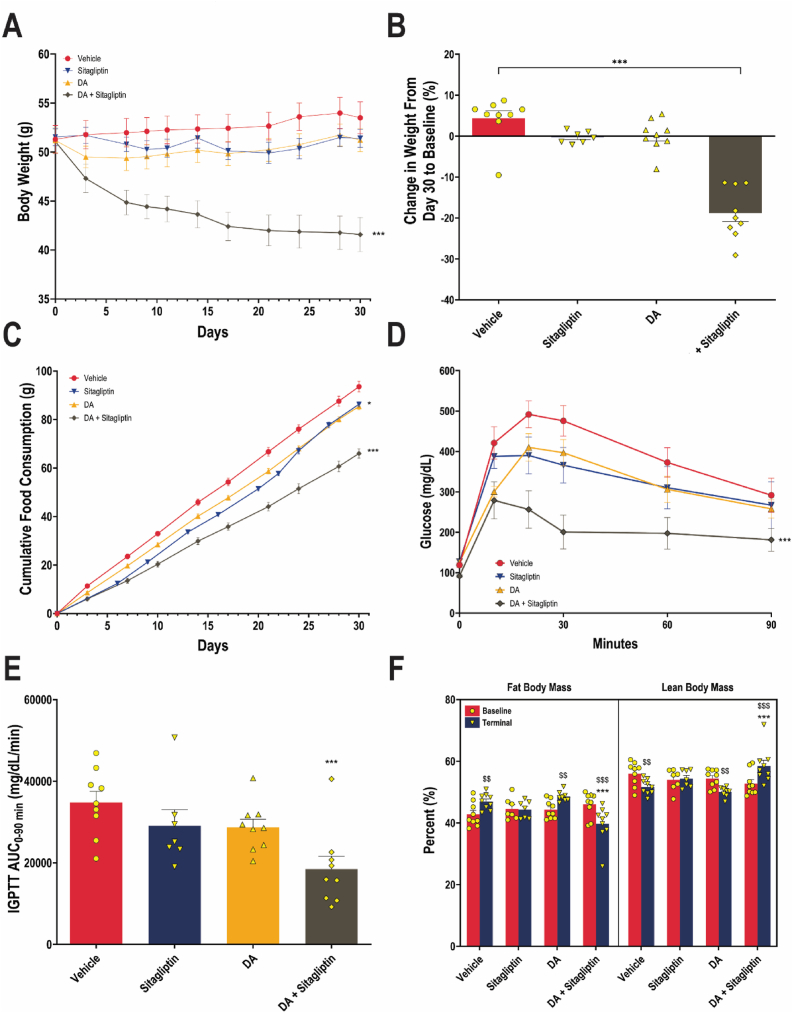
**Body weight changes, cumulative food intake, glucose homeostasis, and body composition in DIO mice treated with DA, sitagliptin-containing HFD, the dual combination, or vehicle.** (A) Body weight trajectories following once-daily oral administration of vehicle (water used as the oral gavage control), DA (75 mg/kg, oral gavage, once daily), 6 g/kg sitagliptin-containing HFD, and the dual combination (75 mg/kg DA, oral gavage, once daily and 6 g/kg sitagliptin-containing HFD). Data are presented as means (dot symbols) ± SEM (bars) for each treatment group. *N* = 9 for vehicle, DA, and dual combination groups. *N* = 7 for sitagliptin-containing HFD group. ∗∗∗*P* < 0.001, vs. the vehicle group at Day 30. (B) Percent changes in body weight from baseline are presented as means (bars) and SEM (error bars), with individual values overlaid (dots). ∗∗∗*P* < 0.001, vs. the vehicle group. (C) Cumulative food intake data are presented as means (dot symbols) ± SEM (bars) for each treatment group. ∗*P* < 0.05 and ∗∗∗*P* < 0.001, vs. the vehicle group at day 30. Glucose curves (D) and calculated AUC_0–90 min_ (E) from IPGTT at Day 14 are presented as means (dot symbols) ± SEM (bars) and means (bars) and SEM (error bars) for each treatment group, respectively. ∗∗∗*P* < 0.001 for AUC_0–90min_ vs. the vehicle group. (F) Percentages of fat and lean body mass were measured at baseline and termination. Data are presented as means (bars) and SEM (error bars), with individual values overlaid (dots). ∗∗∗*P* < 0.001, vs. the vehicle group at the same time point. ^$$^*P* < 0.01, and ^$$$^*P* < 0.001 vs. baseline within the same treatment group.

In Study 2, as shown in Figure 3A, DIO mice co-treated with DA and sitagliptin-containing HFD exhibited reduced body weight trajectories compared to the vehicle-treated controls, DA alone, and sitagliptin-containing HFD groups. Two-way repeated measures ANOVA demonstrated a significant main effect of treatment on body weight (*F* (3, 30) = 8.70, *P* = 0.0003). At the end of the study, body weight increased 4.4% in the vehicle-treated control, remained approximately unchanged in the DA and HFD containing sitagliptin groups, and was significantly reduced by 18.8% (*P* < 0.0001) in mice receiving the combination of DA and sitagliptin-containing HFD (Figure 3B). At the end of the study, cumulative food intake was significantly reduced in DA-treated mice (*P* = 0.012) and more markedly in the combination-treated group (*P* < 0.0001) (Figure 3C). On Day 14, glucose homeostasis, as indicated by glucose AUC_0–90 min_ from intraperitoneal glucose tolerance testing (IPGTT) (Figure 3D,E), was significantly improved in mice receiving DA and sitagliptin-containing HFD, with a significant reduction in AUC_0–90 min_ compared to the vehicle (*P* = 0.0009). At study termination, DEXA analysis revealed that the combination-treated animals exhibited significantly lower fat mass percentage and higher lean mass percentage than the vehicle-treated controls (Figure 3F). These data demonstrate potent synergistic effects of DA and sitagliptin in a preclinical DIO model on reducing food intake and inducing weight loss, along with metabolic improvements in glucose homeostasis and body composition.

### 2.4 Combined treatment with DA and sitagliptin promotes weight loss, reduces food intake, and improves glucose homeostasis and body composition after discontinuation of GLP-1RA in DIO mice

**Figure 4:**
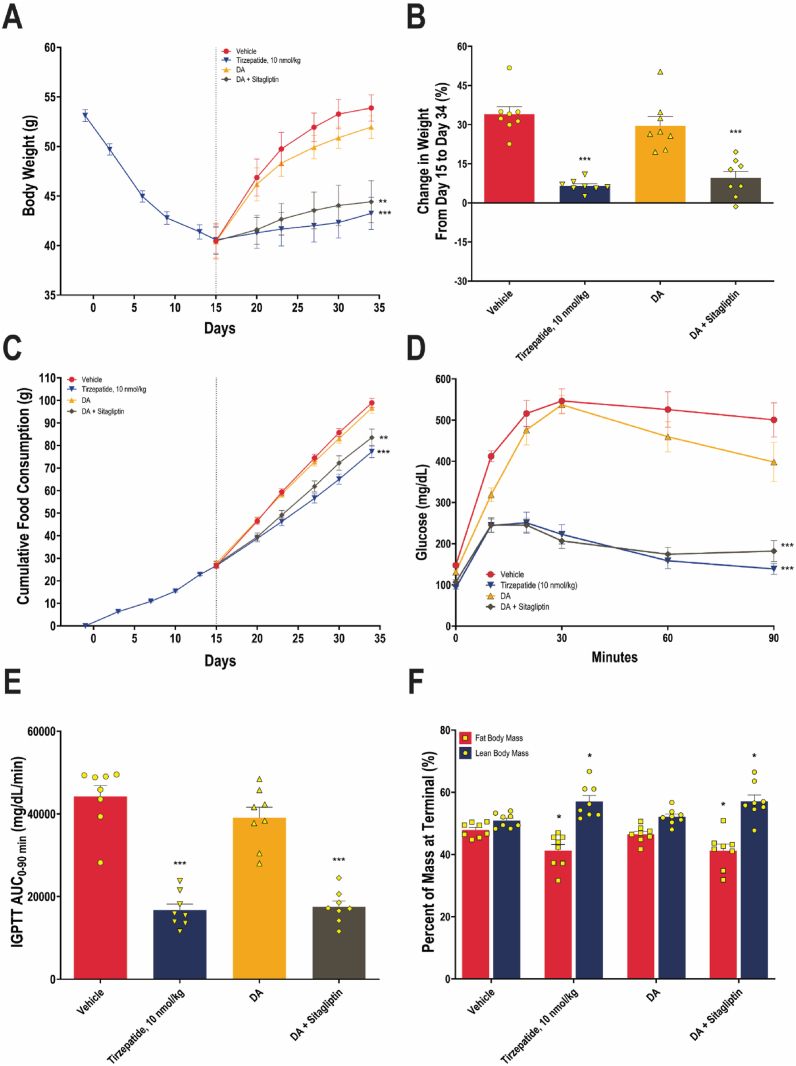
**Body weight changes, cumulative food intake, glucose homeostasis, and body composition in DIO mice treated with tirzepatide, DA, DA and sitagliptin-containing HFD, or vehicle.** (A) Body weight trajectories over two parts. In Part 1, mice were given 10 nmol/kg tirzepatide once daily for two weeks. In Part 2, mice were given either the vehicle (water used as the oral gavage control, and saline used as the subcutaneous injection control), DA (75 mg/kg, oral gavage, once daily), the dual combination (75 mg/kg DA, oral gavage, once daily and 6 g/kg sitagliptin-containing HFD), or continued on 10 nmol/kg tirzepatide for two and a half weeks. Data are presented as means (dot symbols) ± SEM (bars) for each treatment group. *N* = 32 in Part 1; *N* = 8 per group in Part 2. ∗∗*P* < 0.01 and ∗∗∗*P* < 0.001 vs. the vehicle group at Day 34. (B) Percent change in body weight in Part 2 (Days 15–34). Data are presented as means (bars) and SEM (error bars), with individual values overlaid (dots). No statistical significance was identified for the dual combination group vs. the tirzepatide group. ∗∗∗*P* < 0.001, vs. the vehicle group. (C) Cumulative food intake data are presented as means (dot symbols) ± SEM (bars) for each treatment group. ∗∗*P* < 0.01 and ∗∗∗*P* < 0.001 vs. the vehicle group at Day 34. Glucose curves (D) and calculated AUC_0–90 min_ (E) from IPGTT at termination are presented as means (dot symbols) ± SEM (bars) and means (bars) and SEM (error bars) for each treatment group, respectively. ∗∗∗*P* < 0.001 for AUC_0–90 min_ vs. the vehicle group. (F) Percentages of fat and lean body mass were measured at termination. Data are presented as means (bars) and SEM (error bars), with individual values overlaid (dots). ∗*P* < 0.05 vs. the vehicle group.

In Study 3, a two-part DIO mice study was conducted to determine the trajectories of body weight after discontinuation of GLP-1RA treatment. As shown in Figure 4A, in Part 1, mice treated with tirzepatide at 10 nmol/kg exhibited a 12.6 g, or 23.7%, reduction in body weight over a two-week period. Following the cessation of tirzepatide administration, animals in various follow-on treatment groups exhibited divergent body weight trajectories over the subsequent 2.5-week period, with a significant main effect of treatment on body weight (*F* (3, 28) = 5.74, *P* = 0.0034). At the end of Part 2 of the study, body weight gains of 34.0%, 29.6%, 9.5%, and 6.5% were observed in the vehicle, DA, DA and sitagliptin-containing HFD, and tirzepatide (10 nmol/kg) groups, respectively (Figure 4B). There was no statistically significant difference in body weight between those who remained on tirzepatide and those who transitioned to the combination regimen (*P* = 0.852). These data suggest that, after weight loss by GLP-1RA therapy, switching to a combination of DA and sitagliptin can limit weight regain comparably to continued GLP-1RA treatment.

In Part 1 of Study 3, when all the mice were on tirzepatide, the average cumulative food consumption was 27.1 g at Day 15 (Figure 4C). At the end of Part 2, cumulative food intake was significantly reduced, compared to vehicle, in mice co-treated with DA and sitagliptin-containing HFD (*P* = 0.0045), and even more in the tirzepatide group (*P* < 0.0001). However, no statistically significant difference was detected in the cumulative food intake reduction between these two groups (*P* = 0.200). Additionally, glucose homeostasis measured by IPGTT was significantly improved in mice staying on tirzepatide and in those receiving co-treatments of DA and sitagliptin-containing HFD (Figure 4D), evidenced by a significant reduction in AUC_0–90 min_ compared with vehicle (*P* < 0.0001 for all comparisons) (Figure 4E). DEXA analysis also showed that at study termination, the tirzepatide and dual combination groups had a significantly lower fat mass percentage and higher lean mass percentage compared with the vehicle group (Figure 4F). Collectively, these findings highlight the metabolic benefits of the combination of DA and sitagliptin, particularly following GLP-1RA withdrawal.

### 2.5 Oral ARD-101 shows a trend toward body weight control and metabolic improvements in adults with obesity after 28 Days of treatment

In a randomized, single-blind, placebo-controlled, Phase 2 PoC study, 20 adults with obesity (BMI between 30 and 45 kg/m^2^) were treated with either ARD-101 (200 mg, twice daily) or placebo for 28 days. Baseline demographics are summarized in Table 1. ARD-101 was well tolerated. Only two adverse events (AEs) considered related to ARD-101 were reported, both of which were mild in severity (Grade 1): one case of acid reflux and one case of transient nausea. No participants discontinued treatment due to adverse events.

**Table 1:** Demographic and Baseline Characteristics of Pilot Phase 2 Clinical Study of ARD-101 in Adults with Obesity.

Parameter	ARD-101 *N* = 14^a^ (%)	Placebo *N* = 6^a^ (%)	Overall *N* = 20^a^ (%)
**Age**	57 (12) [38, 75]	49 (9) [35, 59]	55 (12) [35, 75]
**Sex**			
Female	10 (71)	3 (50)	13 (65)
Male	4 (29)	3 (50)	7 (35)
**Race**			
Asian	2 (14)	2 (33)	4 (20)
Black or African American	3 (21)	0 (0)	3 (15)
White	9 (64)	4 (67)	13 (65)
**Ethnicity**			
Hispanic or Latino	1 (7.1)	1 (17)	2 (10)
Non-Hispanic or Latino	13 (93)	5 (83)	18 (90)
**Height at baseline (cm)**	167 (9) [157, 187]	169 (11) [159, 188]	168 (10) [157, 188]
**Weight at baseline (kg)**	95.4 (12.4) [80.4, 126.0]	101.6 (23.8) [76.8, 137.4]	97.3 (16.2) [76.8, 137.4]
**BMI at baseline (kg/m^2^)**	34.1 (3.6) [28.5, 38.4]	35.2 (5.5) [29.6, 44.9]	34.5 (4.1) [28.5, 44.9]

BMI, body mass index.

a Mean (SD), [min, max] for continuous variables; n (%) for categorical variables are presented.

**Figure 5:**
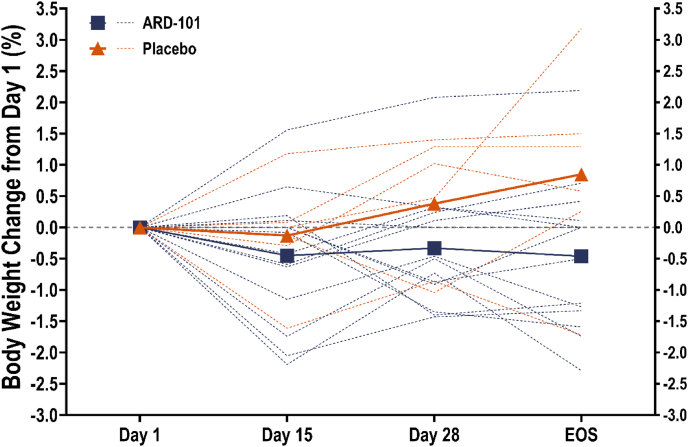
**Effect of ARD-101 on body weight in adults with obesity.** The mean percentage changes in body weight from Day 1 to Day 15, Day 28, and EOS are shown as dot symbols for participants on ARD-101 (blue squares) or placebo (red triangles), with individual values overlaid (dotted lines; blue: participants on ARD-101, red: participants on placebo).

Percent weight change from Day 1 through Days 15 and 28, and EOS (14–21 days post-treatment) was calculated for each participant. As shown in Figure 5, participants in the placebo group exhibited a progressive increase in body weight over time, reaching a mean increase of approximately 0.4% at Day 28 (0.4 ± 1.1% and 0.5 ± 1.1 kg) and 0.8% by EOS (0.8 ± 1.6% and 0.8 ± 1.5 kg). In contrast, participants in the drug-treated group showed an overall decrease in body weight, with a mean change of approximately −0.3% at Day 28 (−0.3 ± 0.9% and −0.3 ± 1.0 kg), which was maintained through the EOS timepoint (−0.5 ± 1.2% and −0.5 ± 1.2 kg).

Changes in lipid parameters from baseline (Run-In, an average of 14.5 days prior to Day 1) to Day 28 are summarized in Supplementary Table 2. Treatment with ARD-101 resulted in a reduction in cholesterol (mean change: −4.4 mg/dL), LDL cholesterol (mean change: −6.8 mg/dL), and non-HDL cholesterol (mean change: −4.6 mg/dL), whereas placebo-treated participants showed a minimal mean change in LDL cholesterol (1.3 mg/dL) and non-HDL cholesterol (1.7 mg/dL), and a slight increase in cholesterol (mean change: 5.7 mg/dL). HDL cholesterol increased in both groups, but the mean rise was slightly lower in the drug-treated group (0.2 mg/dL) compared to placebo (4.0 mg/dL). The total cholesterol to HDL ratio decreased in both groups, reflecting relative improvements in lipid profiles, although this reduction was more pronounced in the placebo group than in the drug group (mean change: −0.2 vs. −0.03). TG levels showed a minimal change in both groups (mean change: −0.6 mg/dL in the ARD-101 group vs. 0.5 mg/dL in the placebo group); the wide inter-subject variability in the drug group (coefficient of variation: 156%) limits definitive interpretation.

ARD-101 treatment showed exploratory metabolic improvements in participants with elevated baseline metabolic parameters. Among four participants with elevated LDL levels (>130 mg/dL, a borderline to moderate cardiovascular risk based on the American College of Cardiology (ACC) and American Heart Association (AHA) guidelines [12]) at baseline, two receiving ARD-101 exhibited substantial reductions in LDL levels between baseline and 28. Specifically, LDL decreased from 161 to 129 mg/dL (19.9% reduction) in one participant and from 178 to 135 mg/dL (24.2% reduction) in another. In contrast, two participants on placebo exhibited minimal changes in LDL-C between those days: one from 134 to 137 mg/dL (2.2% increase) and the other from 145 to 137 mg/dL (5.5% reduction). Additionally, one participant with a baseline (Day 1) HbA1c level of 7.3% experienced a reduction to 6.8% on Day 28 following treatment. These data suggest the potential of ARD-101 to improve dyslipidemia and glycemic control in individuals with obesity and pre-existing metabolic abnormalities.

### 2.6 ARD-101 reduces self-reported hunger feelings and has minimal influence on eating-related positive mood in adults with obesity after 28 Days of treatment

**Figure 6:**
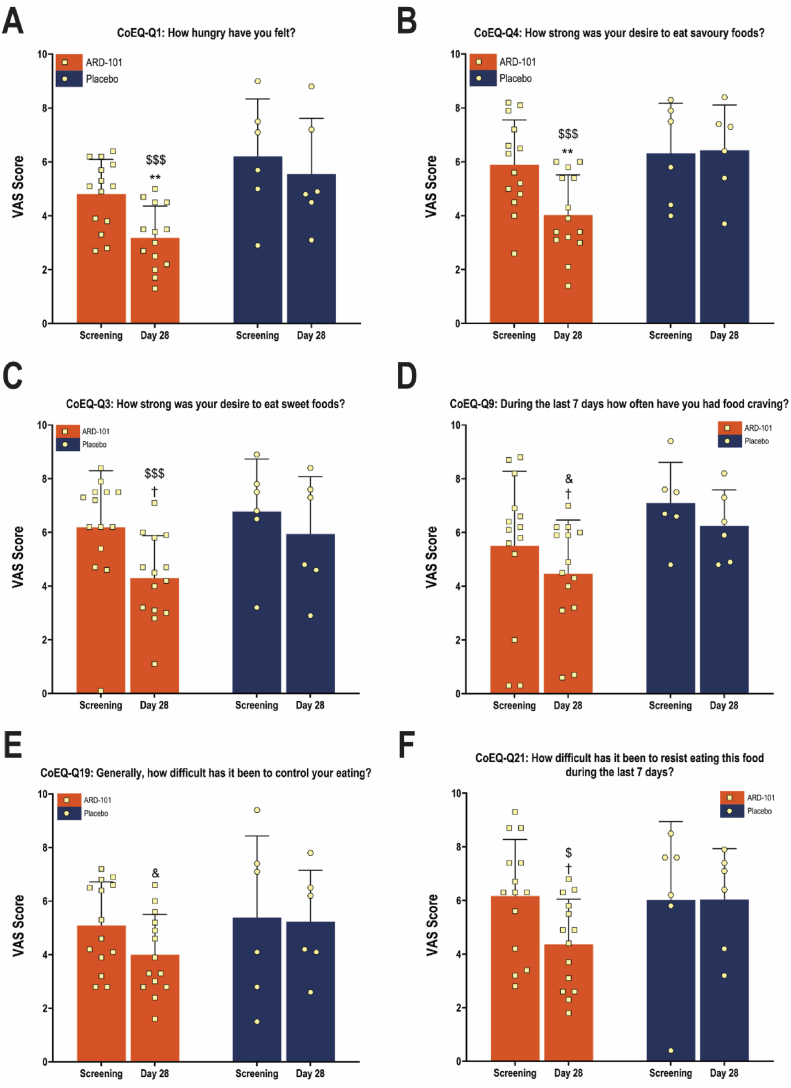
**Effect of ARD-101 on hunger-related scores and exploratory improvement in additional CoEQ domains in adults with obesity following ARD-101 treatment.** VAS ratings of the CoEQ for (A) overall hunger (Question 1), (B) desire to eat savory foods (Question 4), (C) Question 3 (desire to eat sweet foods), (D) Question 9 (frequency of food cravings), (E) Question 19 (difficulty in controlling eating), and (F) Question 21 (difficulty in resisting food triggers) before (at Screening) and after 28 days of treatment (Day 28). Data are shown as means (bars) and SEM (error bars) for ARD-101 (*N* = 14) and placebo (*N* = 6) groups, with individual values overlaid (dots). ∗∗*P* < 0.01, vs. placebo at Day 28. ^&^*P* between 0.05 and 0.1, ^$^*P* < 0.05, and ^$$$^*P* < 0.001 vs. Screening within the ARD-101 group. ^†^*P* between 0.05 and 0.1, vs. placebo at Day 28.

Changes in CoEQ between Screening and Day 28 were assessed and scored on the VAS. As shown in Figure 6A,B, ARD-101 treatment led to significant reductions in VAS scores for CoEQ Question 1 (“How hungry have you felt?”) and Question 4 (“How strong was your desire to eat savory foods?”). Specifically, hunger ratings (Question 1) decreased by 1.63 points in the ARD-101 group versus (vs.) 0.65 points in the placebo group, a 2.5-fold greater reduction. Two-way ANOVA confirmed a significant main effect of treatment for Question 1 (*F* (1, 18) = 7.180 and *P* = 0.0153) and a significant between-group difference at Day 28 (*P* = 0.003). For Question 4, a similar trend was observed (*F* (1, 18) = 4.208 and *P* = 0.055) with a statistically significant difference at Day 28 (*P* = 0.0046).

Beyond the two CoEQ questions described above, four additional CoEQ items (CoEQ Questions 3, 9, 19, and 21) showed directionally favorable improvements with ARD-101 (Figure 6C–F). Notably, for Questions 19 (“Generally, how difficult has it been to control your eating?”) and 21 (“How difficult has it been to resist eating this food during the last 7 days?”) (Figure 6E,F), within-group comparisons revealed noticeable reductions in VAS scores from Screening to Day 28 in the ARD-101 group. In contrast, placebo-treated participants showed minimal change. While two-way ANOVA did not confirm a significant treatment effect across time for these items, the within-group improvements indicate that ARD-101 may influence multiple dimensions of hunger regulation.

For CoEQ items related to eating-related positive mood (Questions 5 (“How happy have you felt?”) and 8 (“How contented have you felt?”)), no significant main effect of treatment with ARD-101 vs. placebo on the change from baseline to Day 28 in VAS scores for these questions was identified, although a significant within-subject variability was observed (Supplementary Fig. 1A and B, Supplementary Table 3). These results suggest that while ARD-101 may reduce hunger, it exerts minimal influence on positive mood aspects of eating behavior and has a limited impact on enjoyment of food.

### 2.7 ARD-101 enhances post-dose GLP-1 and PYY release with trends toward increased CCK and reduced ghrelin in fasted healthy participants

In a double-blind, randomized, placebo-controlled study, healthy participants were treated with ARD-101 (800 mg, once daily) or placebo for two days to assess the effect on gut hormone release. Baseline demographics are summarized in Supplementary Table 4.

**Figure 7:**
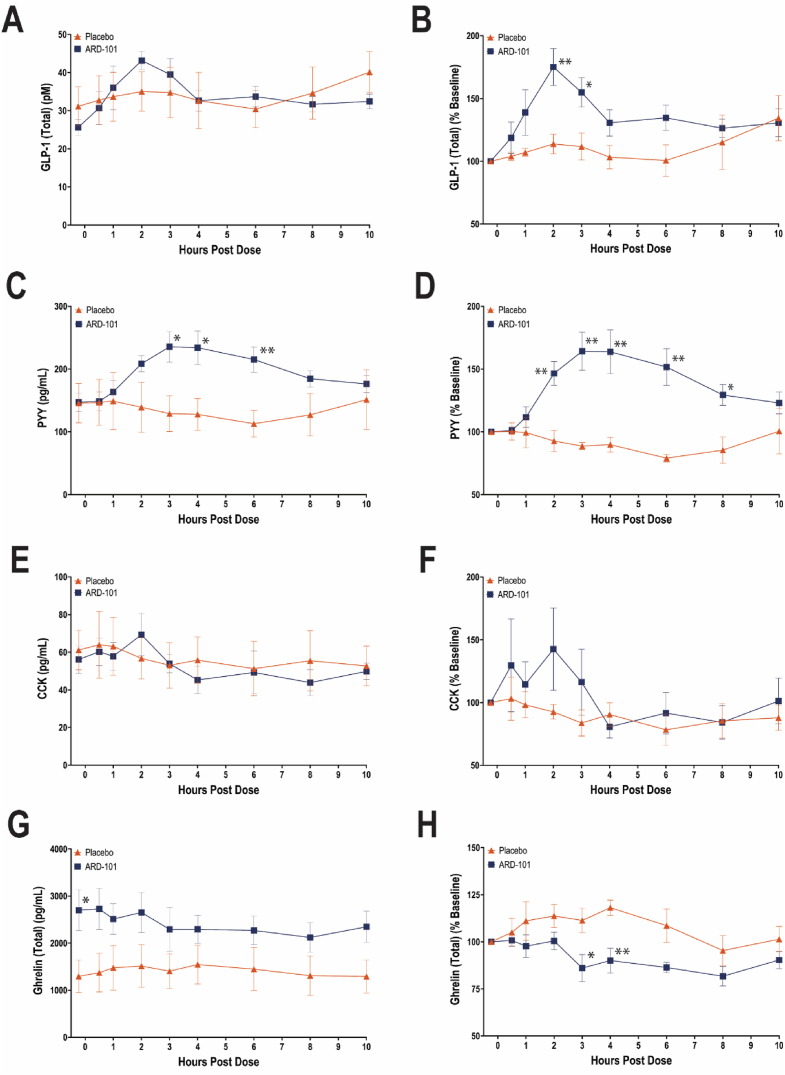
**Effects of ARD-101 on plasma levels of GLP-1 (total), PYY, CCK, and ghrelin (total) in fasted healthy adults.** Absolute plasma concentrations and baseline-normalized percent changes for GLP-1 (total) (A and B), PYY (C and D), CCK (E and F), and ghrelin (total) (G and H) following a single oral dose of ARD-101 at 800 mg. Data are shown as means (bars) and SEM (error bars) for ARD-101 (*N* = 8) and placebo (*N* = 4) groups. ∗*P* < 0.05 and ∗∗*P* < 0.01, vs. placebo at the given time points.

To minimize confounding from food intake and enhance statistical power, analyses of gut hormone responses were limited to Day 2 under fasted conditions, with participants from Cohorts 1 and 2 (eight on ARD-101 and four on placebo, 12 in total) pooled for evaluation. Absolute plasma concentrations at baseline (pre-dose) and up to 10 h post dose, as well as baseline-normalized percent changes for GLP-1 (total), peptide YY (PYY), cholecystokinin (CCK), and ghrelin (total), are presented in Figure 7A–H. Following ARD-101 administration, GLP-1 (total) exhibited a post-dose increase relative to placebo, most evident within the first 3 h post dose (Figure 7A). While no significant main treatment effect was observed for absolute GLP-1 (total) concentrations, baseline-normalized percent changes were significantly greater in ARD-101-treated participants at 2 and 3 h post dose compared with placebo (*P* = 0.0045 and *P* = 0.024, respectively) (Figure 7B). For PYY, ARD-101 treatment resulted in markedly higher plasma concentrations between 3 and 6 h post dose relative to placebo, supported by a significant treatment-by-time interaction (*F*(2.859, 28.59) = 5.259, *P* = 0.0057; Figure 7C), as well as significantly greater baseline-normalized percent increases extending from 2 to 8 h post dose (Figure 7D). In contrast, CCK responses did not demonstrate statistically significant changes in either absolute concentrations or baseline-normalized percent changes; however, a directionally increasing trend within the first 2 h post dose was observed, particularly in the baseline-normalized analysis (Figure 7E,F). For ghrelin (total), ARD-101-treated participants had significantly higher baseline concentrations compared with placebo (*P* = 0.0305; Figure 7G); nonetheless, ARD-101 was associated with a post-dose decreasing trend, most clearly reflected in baseline-normalized percent changes, with significant reductions observed at 3 and 4 h post dose relative to placebo (*P* = 0.0273 and *P* = 0.0047, respectively; Figure 7H).

## 3 Discussion

The present study provides preclinical and early clinical evidence supporting the therapeutic potential of DA, a gut-restricted TAS2R agonist, and its oral drug formulation, ARD-101, currently under clinical development for the treatment of obesity and hyperphagia. In a weight prevention mouse model, twice-daily oral administration of DA over eight weeks led to attenuation of body weight gain, reduced food intake, and beneficial changes in multiple obesity-related metabolic biomarkers. These findings are further supported by a randomized placebo-controlled Phase 2 PoC clinical study in adults living with obesity, in which a 28-day course of orally administered ARD-101 led to a placebo-corrected trend toward weight control and directionally favorable metabolic measurements in participants with elevated baselines, as well as significantly reduced self-reported hunger and selected cravings as assessed by the CoEQ. ARD-101 was generally well tolerated over the 28-day treatment period: two mild, treatment-related adverse events (acid reflux and transient nausea) were reported among ARD-101 recipients, and no participants discontinued for safety reasons.

Treatment with ARD-101 over a 28-day period resulted in an average weight reduction of 0.3 kg, compared to a weight increase of 0.5 kg with placebo. While placebo-treated participants exhibited a progressive increase in weight, weight loss in drug-treated participants was maintained for approximately 16 days following discontinuation, resulting in a placebo-corrected differential of −1.3 kg at EOS. This suggests that the drug’s early effects may persist briefly beyond the dosing period. In the STEP 1 trial evaluating semaglutide 2.4 mg, the placebo-corrected weight loss after 4 weeks was approximately 1.6 kg [13], indicating that the magnitude of effect observed here falls within a comparable range, particularly given the exploratory dosing and brief treatment duration. Importantly, participants in STEP 1 received structured dietary and physical activity counseling, whereas no lifestyle intervention was provided in the present study. This distinction highlights the potential of the investigational drug to elicit weight-lowering effects independently of behavioral support. Furthermore, GLP-1 therapies, such as semaglutide, are known to be associated with rapid weight regain following treatment cessation [14], whereas we did not observe a clear rebound over the short post-treatment follow-up in this trial. The sustained weight effects may reflect differences in mechanism or downstream metabolic responses and raise the possibility of a more stable post-treatment profile. However, head-to-head or adequately powered randomized studies would be needed for any formal comparisons.

The modest changes in lipid parameters over the 28-day treatment period suggest that ARD-101 may exert effects on lipid metabolism. Notably, reductions were seen in LDL cholesterol and non-HDL cholesterol, while HDL cholesterol remained relatively stable. Although triglycerides declined slightly in the drug group, this result was highly variable between individuals, and the mean reduction did not show a consistent pattern. In the context of established weight-loss agents, these lipid changes are broadly consistent with those observed in the early stages of treatment with GLP-1 receptor agonists. For instance, in the STEP 1 trial evaluating semaglutide 2.4 mg, participants experienced significant weight loss accompanied by improvements in cardiometabolic risk factors, including lipid parameters [13]. As mentioned earlier, the current study did not employ dietary or physical activity interventions, in contrast to the structured lifestyle modifications provided in most GLP-1 trials. This distinction may partially account for the more modest changes observed here.

The observed effects are consistent with the proposed mechanism of DA as a gut-restricted TAS2R agonist that modulates hunger-related signaling via TAS2R-driven gut–brain signaling. In the first-in-human (FIH) Phase 1 study, ARD-101 showed minimal systemic exposure [8], indicating a localized action at the gastrointestinal level. This profile is compatible with activation of TAS2Rs on enteroendocrine cells by denatonium, the bitter moiety in ARD-101, and with prior reports that bitter agonists stimulate release of gut peptides such as GLP-1, PYY, and CCK [[15], [16], [17]]. In the Phase 1 study, post-dose elevations in circulating GLP-1 were observed in healthy volunteers [8], and our new data showing increased plasma GLP-1 and PYY levels with a directional trend toward enhanced CCK secretion in healthy participants are consistent with prior reports. Collectively, these findings support a non-nutritive mechanism in which TAS2R activation engages endogenous enteroendocrine signaling pathways without reliance on caloric or nutrient sensing, producing physiological changes in gut peptide release sufficient to influence feeding behavior.

A substantial body of experimental evidence indicates that the satiation and satiety effects of key gut peptides, particularly CCK and GLP-1, are mediated predominantly through activation of vagal afferent pathways rather than through sustained elevations in circulating hormone levels. In rodent models, disruption of vagal afferent signaling markedly attenuates or abolishes CCK- and GLP-1–induced suppression of food intake despite preserved systemic exposure [[18], [19], [20]]. Consistent with these functional findings, enteroendocrine cells form direct, synapse-like connections with vagal sensory neurons, enabling rapid neural transmission of gut signals to the brainstem on a millisecond timescale, prior to endocrine dissemination via the systemic circulation [21,22]. Within this framework, PYY appears to engage both neural and endocrine mechanisms: while circulating PYY can act centrally at Y2 receptors to influence appetite and food intake [23,24], vagal afferent signaling also contributes importantly to physiologic postprandial satiety [25,26]. Together, these observations support a neural-first model of gut–brain communication in which chemosensory activation initiates vagal signaling that is subsequently reinforced, but not driven, by secondary hormone release [22,23]. In contrast to supraphysiologic endocrine delivery of incretin receptor agonists, which relies on sustained systemic exposure and is frequently associated with gastrointestinal intolerance, ARD-101 produces modest, transient changes in circulating gut hormone levels within physiological ranges, indicating that effective suppression of hunger and reinforcement of satiety depend less on absolute hormone concentrations than on coordinated gut–brain signaling that preserves the timing and informational content of enteroendocrine cues.

We emphasize a conceptual distinction between hunger, a homeostatic drive reflecting energy deficit, and appetite, which encompasses hedonic and cue-driven desire to eat. While GLP-1 primarily reinforces the postprandial fed state, delays gastric emptying, and suppresses reward-driven eating [27,28], CCK and PYY are key mediators of meal-induced satiation and inter-meal satiety, shaping both meal termination and post-meal hunger suppression [29,30]. Accordingly, engagement of CCK- and PYY-linked pathways is expected to preferentially modulate hunger and meal termination, whereas GLP-1 contributes primarily to appetite regulation. Findings from the Phase 2 PoC study highlight this pharmacodynamic specificity in humans, where ARD-101 selectively reduced hunger and certain food cravings without altering mood or hedonic drive, consistent with the changes in circulating GLP-1, PYY, CCK, and ghrelin levels. This pattern is particularly relevant in the context of hyperphagia. In individuals with PWS, peripheral CCK secretion is largely preserved but poorly coupled to nutrient sensing, caloric intake, or subjective hunger [[31], [32], [33], [34]], while downstream CCK-responsive circuitry remains recruitable, as demonstrated by suppression of food intake with exogenous CCK in hypothalamic obesity [35]. Elevated circulating ghrelin and attenuated or context-dependent PYY responses may further contribute to persistent hunger in PWS [[36], [37], [38], [39], [40], [41], [42]]. Together, these observations suggest that ARD-101’s engagement of upstream gut nutrient-sensing pathways can improve the coordination and informational content of enteroendocrine signaling, offering a mechanistically plausible approach to reducing pathological hunger without reliance on normalization of any single hormone. ARD-101 is currently being evaluated in clinical studies targeting hyperphagia in patients with PWS (ClinicalTrials.gov: NCT06828861 and NCT07197034).

In a separate DIO model, once-daily DA for four weeks, when combined with a sitagliptin-containing HFD, significantly reduced weight gain, food intake, and obesity-related metabolic measures; these effects were not observed with either DA or sitagliptin alone, indicating combination effects greater than either monotherapy. In Study 2, the dual combination significantly reduced body weight and food intake and improved glucose tolerance, demonstrating synergistic effects in DIO mice. In Study 3, withdrawal of a commonly reported effective dose of tirzepatide (a dual GLP-1/GIP agonist) in these animals led to substantial weight regain, yet DA plus sitagliptin attenuated this regain. Although these experiments were conducted with tirzepatide, the findings may be relevant to incretin receptor agonist-based therapies, for example, semaglutide, where dose-dependent intolerance similarly limits long-term adherence. Mechanistically, for obesity, a condition in which appetite cues and hedonic drive are prominent, pairing DA with DPP-4 inhibition may augment endogenous incretins (e.g., GIP and GLP-1) and thereby enhance overall efficacy. In hunger-predominant syndromes (e.g., PWS), DA monotherapy may be more appropriate, and DPP4 inhibition less favorable due to population-specific risks related to gastrointestinal dysmotility and a high risk of acute gastric dilation, necrosis, and rupture - a known cause of sudden death in PWS.

These findings gain added importance in the context of high real-world discontinuation rates for GLP-1RA therapies and the well-documented clinical consequences of treatment cessation. In a recent U.S. cohort study of 125,474 adults with overweight or obesity, discontinuation of GLP-1RA therapy within one year occurred in 46.5% of those with type 2 diabetes and 64.8% of those without [43]. Discontinuation was driven largely by intolerance, whereas greater weight loss and higher income among patients with diabetes were associated with continued use. Similar patterns are observed outside academic cohorts: a Blue Cross Blue Shield analysis of commercially insured patients reported that fewer than one-third of individuals remained on GLP-1RA therapy at one year, highlighting substantial attrition under real-world conditions [44]. Importantly, discontinuation of GLP-1RA therapy is associated with rapid and substantial weight regain and reversal of metabolic benefits. In the STEP 1 trial extension, participants who discontinued semaglutide regained approximately two-thirds of lost weight within one year, accompanied by deterioration of cardiometabolic risk factors, including glycemic control, blood pressure, and lipid parameters [14]. Substantial weight regain following cessation has been reported with other incretin-based therapies, underscoring the challenge of sustaining benefit with chronic injectable agents. Even under controlled trial conditions, attrition remains substantial: in the SELECT cardiovascular outcomes trial, discontinuation reached approximately 30% overall in the semaglutide group, including 16.6% specifically due to adverse events [45] and in the SURMOUNT-3 phase 3 trial of tirzepatide, discontinuation due to adverse events was reported in 10.5% of participants vs. 2.1% with placebo [46]. Together, these data highlight that intolerance-driven discontinuation is not only common but clinically consequential, motivating the evaluation of alternative or adjunctive approaches - such as ARD-101 with DPP-4 inhibition - that may help sustain metabolic benefit after GLP-1RA discontinuation or provide options for patients unable to remain on incretin-based therapies.

Altogether, our data support ARD-101 as a gut-restricted TAS2R-agonist that blunts hunger and increases satiation/satiety via gut-brain signaling with limited effects on reward-based eating. These features, together with the DA plus sitagliptin findings in established DIO mice, motivate clinical testing in settings where hunger reduction is paramount (e.g., following weight loss and hyperphagic conditions). Nonetheless, this study has several limitations. In the preclinical DIO mouse model, individual daily food intake was not monitored continuously or across light/dark cycles, limiting granularity in feeding behavior analysis, and we cannot rule out that the bitterness impacted food intake. However, measures were taken to minimize this impact on animals; gavage syringes were wiped before administration to remove residual liquid, and dosing was performed more than 4 h before the dark cycle, when most feeding occurs. Mechanistic studies were also not performed, and the precise gut-brain circuits activated by DA remain to be elucidated. Additional studies using peptide antagonists or receptor knockout models, as well as a comprehensive analysis of gut peptides in animals started on HFD vs. those with established DIO, could help clarify the gut peptide pathways underlying DA’s mechanism of action. As to the clinical data, the Phase 2 PoC study was limited by small sample size without powering for weight loss endpoints, single-center design, and short duration. Larger, long-term studies are needed to assess the durability, safety, and efficacy of ARD-101 across diverse patient populations.

In conclusion, DA and its oral drug formulation ARD-101 are a promising investigational approach for the treatment of hyperphagia, including conditions such as PWS, leveraging a novel gut-restricted mechanism to reduce hunger and increase satiation/satiety via TAS2R-mediated gut-brain signaling while avoiding broad suppression of enjoyment of food. The preclinical and early clinical data presented here support the continued evaluation of ARD-101 in broader and long-term studies, addressing unmet needs in the current hyperphagia treatment landscape. Given ARD-101’s primary effect on hunger and satiety, future studies should enroll cohorts undergoing caloric restriction or with hyperphagia-predominant conditions to test whether hunger attenuation translates to greater adherence and weight outcomes; for obesity, ARD-101 with DPP-4 inhibition should also be evaluated.

## 4 Materials and methods

### 4.1 Animals and reagents

Study 1: Twelve-week-old male C57BL/6NTac mice were purchased from Taconic Biosciences, Inc. (Rensselaer, NY). Upon arrival, animals were weighed using an electronic balance (Ohaus SCOUT® PRO, Parsippany, NJ), underwent a clinical health assessment, and were group-housed (up to three per cage) in High Efficiency Particulate Air (HEPA)-filtered static cages containing Teklad™ Sani-Chips bedding (7090A; Inotiv, Inc., West Lafayette, IN). Environmental conditions were maintained at 22 °C ± 4 °C with 50% ± 20% relative humidity under a 12-hour light/dark cycle. Animals were acclimated for a minimum of three days prior to study initiation. The mice arrived on a standard chow diet (2018 Teklad Global 18% Protein, Inotiv, Inc., West Lafayette, IN) and were maintained with ad libitum access to food and water. They were transitioned to an HFD (60% kcal from fat; Research Diets D12492, Research Diets, Inc., New Brunswick, NJ) and maintained on HFD for 8 days prior to study initiation, after which mice were housed individually and had ad libitum access to water and the HFD, except during designated fasting periods. Based on the duration of HFD feeding and the body weight of the vehicle group at the end of study, mice exhibited a phenotype consistent with DIO at termination.

Study 2 and Study 3: Twenty-two-week-old male C57BL/6NTac mice were purchased from Taconic Biosciences, Inc. They were maintained on HFD prior to and following arrival, thereby generating DIO. Upon arrival, animals were handled and manipulated in the same manner as in Study 1. Mice were acclimated for 2–5 weeks with ad libitum access to water and HFD prior to study initiation. The mice were individually housed throughout the study and had ad libitum access to water and either HFD or a custom-formulated HFD by Research Diets Inc. to include 6 g of sitagliptin (Adooq Bioscience, Irvine, CA; purity >99%) per kilogram of HFD except during designated fasting periods. This sitagliptin dose represents a middle-range level consistent with sitagliptin diet formulations reported in murine models [[47], [48], [49]].

Denatonium acetate (DA, purity >99% by HPLC) was provided by Aardvark Therapeutics and stored at room temperature until formulation. In Study 1, weekly dosing formulations were freshly prepared by dissolving DA in distilled water (vehicle) to achieve final concentrations of 4, 8, and 16 mg/mL, corresponding to dose levels of 20, 40, and 80 mg/kg based on the active bitter moiety. In Studies 2 and 3, dosing formulations were prepared as in Study 1, to achieve a final concentration of 7.5 mg/mL, corresponding to a dose level of 75 mg/kg based on the active bitter moiety.

In Study 3, tirzepatide (Adooq Bioscience, Lot No. L20956B001) was formulated in 0.9% sterile saline for subcutaneous dosing to achieve a final concentration of 10 nmol/mL, corresponding to a dose level of 10 nmol/kg. The 10 nmol/kg dose is within the established effective weight-control range for murine models [50]. New dosing solutions were prepared weekly from aliquots of frozen stock solutions, thawed and mixed immediately before dosing, and stored at 4 °C when not in use.

### 4.2 Animal grouping, dosing regimen, and sample collection procedures

At the initiation of Study 1, all animals were weighed and randomized into treatment groups (*N* = 12 per group) to ensure comparable body weight distribution across cohorts. Groups included a vehicle control and three DA treatment arms (20, 40, and 80 mg/kg). The test article was administered via oral gavage twice daily at 5 mL/kg for a total of eight weeks. The selected dose levels and frequency were aligned with clinically relevant dose regimens.

In Study 2, all animals were weighed and randomized into four treatment groups (*N* = 7–9 per group): vehicle control (water used as the oral gavage control), DA (75 mg/kg) on standard HFD, vehicle on sitagliptin HFD, and DA (75 mg/kg) on sitagliptin-containing HFD. DA was administered once daily via oral gavage at 10 mL/kg, and sitagliptin-containing HFD was available ad libitum for 4 weeks.

At the initiation of Phase 1 of Study 3, all mice (*N* = 32) received tirzepatide (10 nmol/kg) administered subcutaneously at 1 mL/kg once daily for 2 weeks, while they were maintained on a standard HFD. Unexpectedly, two separate preparations of tirzepatide (Selleck Chemicals, Houston, TX; Lot No. P120601; Adooq Biosciences, Lot No. L20956B003) failed to induce weight loss. A lack of biological activity for this tirzepatide was confirmed in cell-based bioassays (data not shown). In contrast, a third preparation (Adooq Biosciences, Lot No. L20956B001) produced weight loss consistent with prior experiences in this model and was utilized for the study. In Phase 2, animals were randomized into four treatment groups: vehicle control (water used as the oral gavage control, and saline used as the subcutaneous injection control), tirzepatide (10 nmol/kg), DA (75 mg/kg), and DA (75 mg/kg) plus sitagliptin-containing HFD. Animals transitioned from standard HFD to sitagliptin-containing HFD at the start of Phase 2, where applicable. Tirzepatide was administered subcutaneously once daily at 1 mL/kg, DA was delivered via oral gavage once daily at 10 mL/kg, and all diets were provided ad libitum for 2.5 weeks.

In Studies 1–3, body weight was recorded two to three times weekly from the first day of dosing. In Study 1, food intake was assessed over a 24-hour period for each animal on Day 1 and subsequently on Days 14, 27, and 42 (corresponding to Weeks 2, 4, and 6). Blood samples were collected at baseline (pre-dose) and at study termination (Week 8) following a ∼6-hour fasting period. Whole blood was analyzed immediately for glucose, while the remaining volume was processed to obtain serum for metabolic biomarker analysis. To adhere to ethical blood collection limits, baseline serum samples were obtained from a randomly selected subset of six mice per group, with each subset allocated to measure two or three of the five predefined biomarkers. In Studies 2 and 3, cumulative food intake was measured every 3–4 days. Blood samples were collected at baseline (pre-dose) and at study termination after an approximately 5-hour fasting period. Fasting IPGTT was performed at the midpoint of Study 2, and at the conclusion of Study 3.

All animal procedures were approved by the Institutional Animal Care and Use Committee (IACUC) under the Office of Laboratory Animal Welfare (OLAW) Assurance No. A457301.

### 4.3 Metabolic biomarker measurements

Fasting blood glucose levels were measured immediately after collection using a handheld glucometer (True Metrix®, Trividia Health, Inc., Fort Lauderdale, FL). Serum biomarkers were quantified using validated commercial assay kits, following the manufacturers’ protocols with included calibrators and controls. Insulin concentrations were measured using the Ultra-Sensitive Mouse Insulin ELISA Kit (Crystal Chem, Elk Grove Village, IL; Catalog No. 90080). Low-density lipoprotein (LDL) cholesterol was assessed using the LDL Cholesterol Mouse Assay Kit (Crystal Chem; Catalog No. 79980), and triglycerides (TG) were measured using the Triglyceride Assay Kit (Abcam, Cambridge, UK; Catalog No. ab65336). Total cholesterol (TC) was measured using the Cholesterol/Cholesteryl Ester Assay Kit (BioVision, Milpitas, CA; Catalog No. K603-100).

### 4.4 Glucose tolerance testing

Mice were fasted for approximately 4 h with free access to water prior to IPGTT. Each animal was weighed to calculate the required 20% glucose dose (2 g/kg) for intraperitoneal injection at 10 μL/g body weight. A small tail nick was performed under topical anesthetic to obtain baseline blood glucose (*t* = 0), measured with a True Metrix® glucometer (Trividia Health, Inc., Fort Lauderdale, FL; upper limit 600 mg/dL). Mice were then injected intraperitoneally with the calculated glucose dose, and blood glucose was measured at 0, 10, 20, 30, 60, and 90 min post-injection using small tail blood drops. Between measurements, bleeding was minimized by gentle pressure, and at the end of the study, mice were returned to clean cages with food and water. Animals were monitored throughout for normal recovery. Area-under-the-curve (AUC) for glucose was calculated using the trapezoidal rule.

### 4.5 Whole-body composition measurements

Dual-energy X-ray absorptiometry (DEXA) was performed using an InAlyzer 2 system (Medikors, Inc., Seongnam, Republic of Korea) to quantify the percentages of whole-body fat and lean mass. Mice were anesthetized with ketamine/xylazine (ketamine: 80–100 mg/kg; xylazine: 10–15 mg/kg, intramuscular) and positioned prone with limbs gently extended on the scanner bed without restraints. Automated tissue segmentation and data analysis were performed using the InAlyzer software, and results were expressed as percentages of body weight. Prior to each scanning session, system calibration was conducted using the manufacturer’s standard phantom according to recommended quality control procedures.

### 4.6 Overview of clinical studies

A single-blinded, randomized, placebo-controlled, Phase 2 PoC clinical study (Clinical Study 1) was conducted at the Altman Clinical and Translational Research Institute, University of California (UC) San Diego, to evaluate the translational relevance of DA in adults with obesity. All participants provided written informed consent prior to enrollment. The study protocol was approved by the UC San Diego Institutional Review Board (IRB) and adhered to the Declaration of Helsinki. The study is registered on ClinicalTrials.gov (NCT05121441); full eligibility criteria and study endpoints are available on the registry.

Participants were randomly assigned to receive the oral drug formulation of DA, ARD-101 (200 mg denatonium base, twice daily), or a matching placebo (containing the same inactive excipients as ARD-101 but lacking DA) for 28 days. The dosing regimen was identical across treatment arms. Body weight was measured at baseline (Day 1), Day 15, Day 28 (end of treatment), and end of study (EOS). Study endpoints, including metabolic markers, were assessed at baseline (Run-In for blood lipids and Day 1 for glycated hemoglobin (HbA1c)) and at the end of the treatment period (Day 28). Changes in eating behavior were assessed using the 21-item CoEQ (Supplementary Table 5) [51], administered at baseline (Screening) and Day 28. Each item was scored on a visual analog scale (VAS) ranging from 0 (“not at all”) to 10 (“extremely”), capturing changes in hunger, cravings, and eating control.

A double-blind, randomized, placebo-controlled, exploratory clinical pharmacology study (Clinical Study 2) was conducted at Quotient Sciences in Nottingham, United Kingdom (UK) to evaluate the effects of ARD-101 on gut hormones in humans. Healthy adult participants were enrolled in accordance with protocol-defined eligibility criteria. The study was conducted in adherence to the Declaration of Helsinki and Good Clinical Practice. Written informed consent was obtained from all participants prior to study participation, and the protocol and informed consent form were approved by an independent ethics committee. This study is registered on the Integrated Research Application System (IRAS) in the UK (No. 1011885).

The study employed a two-cohort design to assess pharmacodynamic responses under controlled fed and fasted conditions. On Day 1, cohorts differed with respect to meal timing. In Cohort 1, participants consumed a standardized breakfast (562 kcal with 25% energy from fat) shortly after the morning baseline blood draw, followed by dosing approximately 2 h later. In Cohort 2, participants were dosed under fasted conditions, with the standardized breakfast administered approximately 2 h post dose. On Day 2, all participants in both cohorts were dosed under similar fasted conditions, and pharmacodynamic sampling was timed relative to the Day 2 dose; no meal was administered during the Day 2 sampling period.

Six participants were enrolled in each cohort. Using a computer-generated randomization schedule, participants were assigned to receive ARD-101 (800 mg denatonium base, once daily) or a matching placebo (containing the same inactive excipients as ARD-101, but lacking DA) at a 4:2 ratio within each cohort.

Blood samples were collected into BD™ P800 tubes (BD Biosciences, Franklin Lakes, NJ) at predefined time points before and after dosing and were maintained under refrigerated conditions, centrifuged to separate plasma, and stored frozen until analysis. Plasma concentrations of GLP-1 (total), PYY, CCK, and ghrelin (total) were measured using electrochemiluminescence assays with an MSD SQ120 instrument (Meso Scale Discovery, Rockville, MD) in accordance with the manufacturer’s instructions. For all assays, 25 μL of plasma was analyzed per well; assays were performed in duplicate, and the mean of the duplicate measurements was reported for each analyte. For samples in which one replicate was below blank, the value from the remaining replicate was reported.

### 4.7 Statistical analysis

All statistical analyses were conducted using GraphPad Prism software (version 10.4.1; GraphPad Software Inc., Boston, MA). Continuous variables are reported as means ± standard error of the mean (SEM), and categorical variables as counts and percentages. One-way analysis of variance (ANOVA) was used for multi-group comparisons at single time points. For repeated measures, two-way ANOVA was used when complete datasets were available. In cases with missing values or time points with partial data, a mixed-effects model was applied to account for incomplete datasets.

In the animal studies, multiple comparisons across time points and between groups were adjusted using the two-stage linear step-up procedure of Benjamini, Krieger, and Yekutieli (BKY method) to control the false discovery rate. For CoEQ outcomes, as well as plasma concentrations and baseline-normalized percent changes in gut hormones, the Fisher’s Least Significant Difference (LSD) method was used without correction to increase sensitivity for detecting potential treatment effects.

Statistical significance was defined as a two-sided *P* value < 0.05. Values between 0.05 and 0.1 were considered indicative of a trend and potentially meaningful for future investigation.

## CRediT authorship contribution statement

**Zhenhuan Zheng:** Writing – review & editing, Writing – original draft, Visualization, Validation, Supervision, Software, Methodology, Investigation, Formal analysis, Data curation. **Jeremy H. Pettus:** Supervision, Investigation. **Alexa Warner:** Project administration, Data curation. **Bryan Jones:** Writing – review & editing, Supervision, Project administration. **Megan Pugsley:** Writing – review & editing, Writing – original draft, Visualization, Software, Investigation, Formal analysis, Data curation. **Justin Stege:** Writing – review & editing, Writing – original draft, Supervision, Project administration, Methodology, Investigation. **Brad Hirakawa:** Writing – review & editing, Writing – original draft, Visualization, Software, Formal analysis, Data curation. **Manasi Jaiman:** Writing – review & editing, Validation, Supervision. **Jerlyn Tolentino:** Project administration, Supervision. **Tien-Li Lee:** Supervision, Resources, Methodology, Funding acquisition, Conceptualization. **Timothy J. Kieffer:** Writing – review & editing, Writing – original draft, Supervision, Resources, Methodology, Conceptualization.

## Declaration of generative AI and AI-assisted technologies in the manuscript preparation process

During the preparation of this work, the authors used ChatGPT 5.2 in order to correct and improve the readability and language of the manuscript. After using this tool/service, the author(s) reviewed and edited the content as needed and take(s) full responsibility for the content of the published article.

## Funding

None to disclose.

## Declaration of competing interest

Zhenhuan Zheng, Alexa Warner, Bryan Jones, Megan Pugsley, Justin Stege, Brad Hirakawa, Manasi Jaiman, Jerlyn Tolentino, Tien-Li Lee, and Timothy J. Kieffer are full-time employees of Aardvark Therapeutics, Inc. Jeremy H. Pettus has received Study Principal Investigator fees from Aardvark Therapeutics, Inc.

## Data Availability

Data will be made available on request.
