# Supplementary material for: ARD-101, a gut-restricted TAS2R agonist, reduces hunger in adults and promotes weight loss in DIO mice with DPP-4 inhibition
Source: Mol Metab. 2026 Mar 14;106:102340. doi: 10.1016/j.molmet.2026.102340 (PMC12990342; doi:10.1016/j.molmet.2026.102340)
Supplement: Multimedia component 1 [file mmc1.docx]

ARD-101, a Gut-Restricted TAS2R Agonist, Reduces Hunger in Adults and Promotes Weight Loss in DIO Mice with DPP-4 Inhibition

Zhenhuan Zheng^1^, Jeremy H. Pettus^2^, Alexa Warner^1^, Bryan Jones^1^, Megan Pugsley^1^, Justin Stege^1^, Brad Hirakawa^1^, Manasi Jaiman^1^, Jerlyn Tolentino^1^, Tien-Li Lee^1^, and Timothy J. Kieffer^1,3^

*1. Aardvark Therapeutics, 4370 La Jolla Village Drive, Suite 1050, San Diego, CA 92122, USA.*

*2. Division of Endocrinology, Altman Clinical and Translational Research Institute (ACTRI), University of California, San Diego, 9452 Medical Center Drive L1W-515, La Jolla, CA 92037, USA.*

*3. Life Sciences Institute, Department of Cellular and Physiological Sciences, Department of Surgery, School of Biomedical Engineering, University of British Columbia; Vancouver BC, V6T1Z3, Canada*

Corresponding Author: Timothy J. Kieffer, PhD, FCAHS

Email: [tim.kieffer@aardvarktherapeutics.com](mailto:tim.kieffer@aardvarktherapeutics.com)

Tel: +1-(858) 225-7696

Fax: +1-(858) 408-4448

ORCID ID: 0000-0003-4548-3176

Name and address of author to whom reprint requests should be addressed

Timothy J. Kieffer, PhD, Aardvark Therapeutics, 4370 La Jolla Village Drive, Suite 1050, San Diego, CA 92122, USA

ClinicalTrials.gov number: NCT05121441

Integrated Research Application System (IRAS) number: 1011885

**Supplementary Information**

**Supplementary Table 1. Mixed-Effects Analysis Showing Significant Effects of DA on Metabolic Biomarkers in DIO Mice**

| **Metabolic Biomarker** | **Fixed Effects** | | | | **Random Effects** | | |
| --- | --- | --- | --- | --- | --- | --- | --- |
|  | **Statistical Parameter** | **Time** | **Treatment** | **Time × Treatment** | **Statistical Parameter** | **Animal** | **Residual** |
| **Glucose** | ***P* Value** | < 0.0001 | 0.0138 | 0.0696 | **SD** | 0.000 | 23.38 |
|  | ***F* Value**  **(DFn, DFd)** | *F* (1,64) =21.91 | *F* (3,64) =3.829 | *F* (3,64) =2.472 | **Variance** | 0.000 | 546.4 |
| **Insulin** | ***P* Value** | < 0.0001 | 0.0074 | < 0.0001 | **SD** | 3.395 | 2.008 |
|  | ***F* Value**  **(DFn, DFd)** | *F* (1,20) =70.59 | *F* (3,44) =4.539 | *F* (3,20) =12.06 | **Variance** | 11.52 | 4.034 |
| **LDL** | ***P* Value** | <0.0001 | 0.0004 | 0.5127 | **SD** | 16.21 | 262.7 |
|  | ***F* Value**  **(DFn, DFd)** | *F* (1,20) =112.5 | *F* (3,44) =7.533 | *F* (3,20) =0.7918 | **Variance** | 30.18 | 910.8 |
| **TG** | ***P* Value** | 0.0027 | <0.0001 | <0.0001 | **SD** | 1.799 | 3.350 |
|  | ***F* Value**  **(DFn, DFd)** | *F* (1,20) =11.71 | *F* (3,44) =36.62 | *F* (3,20) =43.81 | **Variance** | 3.235 | 11.22 |
| **TC** | ***P* Value** | <0.0001 | <0.0001 | <0.0001 | **SD** | 0.000 | 26.11 |
|  | ***F* Value**  **(DFn, DFd)** | *F* (1,63) =117.2 | *F* (3,63) =9.686 | *F* (3,63) =10.93 | **Variance** | 0.000 | 681.6 |

Significance was determined at α = 0.05.

LDL, low-density lipoprotein; TG, triglycerides; TC, total cholesterol; DFn, Degrees of Freedom Numerator; DFd, Degrees of Freedom Denominator; SD, standard deviation

**Supplementary Table 2. Changes in Blood Lipids on Day 28 from Baseline (Run-in) in Adults with Obesity in Clinical Study 1**

| **Lipid** | **ARD-101 (n = 14) *** | **Placebo (n = 6) *** |
| --- | --- | --- |
| Cholesterol (mg/dL) | -4.4 (17.3), [-53, 18] | 5.7 (19.3), [-11, 42] |
| Cholesterol/HDL-Cholesterol | -0.03 (0.6), [-1.0, 0.9] | -0.2 (0.5), [-0.7, 0.5] |
| HDL Cholesterol (mg/dL) | 0.2 (7.4), [-8, 13] | 4.0 (6.1), [-7, 11] |
| LDL Cholesterol (mg/dL) | -6.8 (17.0), [-43, 11]^$^ | 1.3 (15.2), [-13, 30] |
| Non-HDL Cholesterol (mg/dL) | -4.6 (18.2), [-50, 14] | 1.7 (17.8), [-16, 36] |
| Triglycerides (mg/dL) | -0.6 (93.8), [-233, 221] | 0.5 (27.6), [-29, 40] |

* Mean (SD), [Min, Max]

^$^ n = 13 due to one missing value for LDL Cholesterol

**Supplementary Table 3. Two-way ANOVA Analysis of CoEQ Questions 5 and 8**

| ***CoEQ-Q5 (“How happy have you felt?”) - Screening and Day 28*** | | | | | | |
| --- | --- | --- | --- | --- | --- | --- |
| **ANOVA table** | **% of total variation** | **SS** | **DF** | **MS** | **F (DFn, DFd)** | ***P* value** |
| Time x Treatment | 2.966 | 3.895 | 1 | 3.895 | F (1, 18) = 4.142 | *P*=0.0568 |
| Time | 0.5666 | 0.7440 | 1 | 0.7440 | F (1, 18) = 0.7913 | *P*=0.3855 |
| Treatment | 0.1439 | 0.1890 | 1 | 0.1890 | F (1, 18) = 0.03178 | *P*=0.8605 |
| Subject | 81.53 | 107.1 | 18 | 5.948 | F (18, 18) = 6.325 | *P*=0.0001 |
| Residual |  | 16.93 | 18 | 0.9403 |  |  |
| ***CoEQ-Q8 (“How contented have you felt?”) - Screening and Day 28*** | | | | | | |
| **ANOVA table** | **% of total variation** | **SS** | **DF** | **MS** | **F (DFn, DFd)** | ***P* value** |
| Time x Treatment | 2.531 | 4.200 | 1 | 4.200 | F (1, 18) = 2.759 | *P*=0.1141 |
| Time | 0.8391 | 1.392 | 1 | 1.392 | F (1, 18) = 0.9144 | *P*=0.3516 |
| Treatment | 0.4201 | 0.6972 | 1 | 0.6972 | F (1, 18) = 0.09737 | *P*=0.7586 |
| Subject | 77.66 | 128.9 | 18 | 7.160 | F (18, 18) = 4.702 | *P*=0.0010 |
| Residual |  | 27.41 | 18 | 1.523 |  |  |

SS, Sum of Squares; MS, Mean Square; DF, Degrees of Freedom; DFn, Degrees of Freedom Numerator; DFd, Degrees of Freedom Denominator

**Supplementary Table 4. Baseline Demographics of Enrolled Healthy Adult Participants in Clinical Study 2**

|  | **Cohort 1** | | **Cohort 2** | | **Overall** | | |
| --- | --- | --- | --- | --- | --- | --- | --- |
| **Parameter** | **ARD-101** | **Placebo** | **ARD-101** | **Placebo** | **ARD-101** | **Placebo** | **Total** |
|  | **N=4^1^ (%)** | **N=2^1^ (%)** | **N=4^1^ (%)** | **N=2^1^ (%)** | **N=8^1^ (%)** | **N=4^1^ (%)** | **N=12^1^ (%)** |
| **Age** | 39 (7),  [31, 48] | 42 (2),  [40, 43] | 34 (6),  [25, 39] | 40 (1),  [39, 41] | 36 (7),  [25, 48] | 41 (2),  [39, 43] | 38 (6),  [25, 48] |
| **Sex** |  |  |  |  |  |  |  |
| Female | 1 (25) | 0 | 1 (25) | 0 | 2 (25) | 0 | 2 (17) |
| Male | 3 (75) | 2 (100) | 3 (75) | 2 (100) | 6 (75) | 4 (100) | 10 (83) |
| **Race** |  |  |  |  |  |  |  |
| Asian | 1 (25) | 0 (0) | 2 (50) | 0 (0) | 3 (37.5) | 0 (0) | 3 (25) |
| Black or African American | 0 (0) | 0 (0) | 0 (0) | 0 (0) | 0 (0) | 0 (0) | 0 (0) |
| White | 3 (75) | 2 (100) | 2 (50) | 2 (100) | 5 (62.5) | 4 (100) | 9 (75) |
| **Ethnicity** |  |  |  |  |  |  |  |
| Hispanic or Latino | 0 (0) | 0 (0) | 0 (0) | 0 (0) | 0 (0) | 0 (0) | 0 (0) |
| Non-Hispanic or Latino | 4 (100) | 2 (100) | 4 (100) | 2 (100) | 8 (100) | 4 (100) | 12 (100) |
| **Height at Baseline (cm)** | 174 (8), [163, 182] | 173 (1), [172, 174] | 170 (6), [162, 176] | 172 (8), [166, 177] | 172 (7), [162, 182] | 172 (5), [166, 177] | 172 (6), [162, 182] |
| **Weight at Baseline (kg)** | 76.6 (12.2),  [58.9, 85.3] | 88.9 (5.2),  [85.2, 92.6] | 69.1 (13.1),  [55.7, 86.2] | 88.3 (1.0),  [87.6, 89.0] | 72.9 (12.4),  [55.7, 86.2] | 88.6 (3.1),  [85.2, 92.6] | 78.1 (12.7),  [55.7, 92.6] |
| **BMI at Baseline (kg/m^2^)** | 25.2 (2.7),  [22.2, 27.5] | 29.7 (2.3),  [28.1, 31.3] | 23.7 (2.9),  [21.2, 27.8] | 30.1 (2.4),  [28.4, 31.8] | 24.4 (2.7),  [21.2, 27.8] | 29.9 (1.9),  [28.1, 31.8] | 26.3 (3.6),  [21.2, 31.8] |

^1^Mean (SD), [min, max] for continuous variables; n (%) for categorical variables are presented.

BMI, body mass index

**Supplementary Table 5. Control of Eating Questionnaire**

| Instructions: Please read each question carefully and put a mark through the line at the point that best represents your experience. Answer all questions according to your experience over the **last 7 days**. |
| --- |
| 1. How hungry have you felt?  2. How full have you felt?  3. How strong was your desire to eat sweet foods?  4. How strong was your desire to eat savory foods? |
| 5. How happy have you felt?  6. How anxious have you felt?  7. How alert have you felt?  8. How contented have you felt? |
| A food craving is a strong urge to eat a particular food or drink  9. During the last 7 days how often have you had food cravings?  10. How strong have any food cravings been?  11. How difficult has it been to resist any food cravings?  12. How often have you eaten in response to food cravings? |
| How often have you had food cravings for the following types of food/drink?  13. Chocolate or chocolate flavored foods  14. Other sweet foods (cakes, pastries, biscuits, etc.)  15. Fruit or fruit juice  16. Dairy foods (cheese, yoghurts, milk, etc.)  17. Starchy foods (bread, rice, pasta, etc.)  18. Savory foods (French fries, crisps, burgers, pizza, etc.) |
| 19. Generally, how difficult has it been to control your eating?  20. Which one food makes it most difficult for you to control eating?  21. How difficult has it been to resist eating this food during the last 7 days? |

**Supplementary Figure 1**


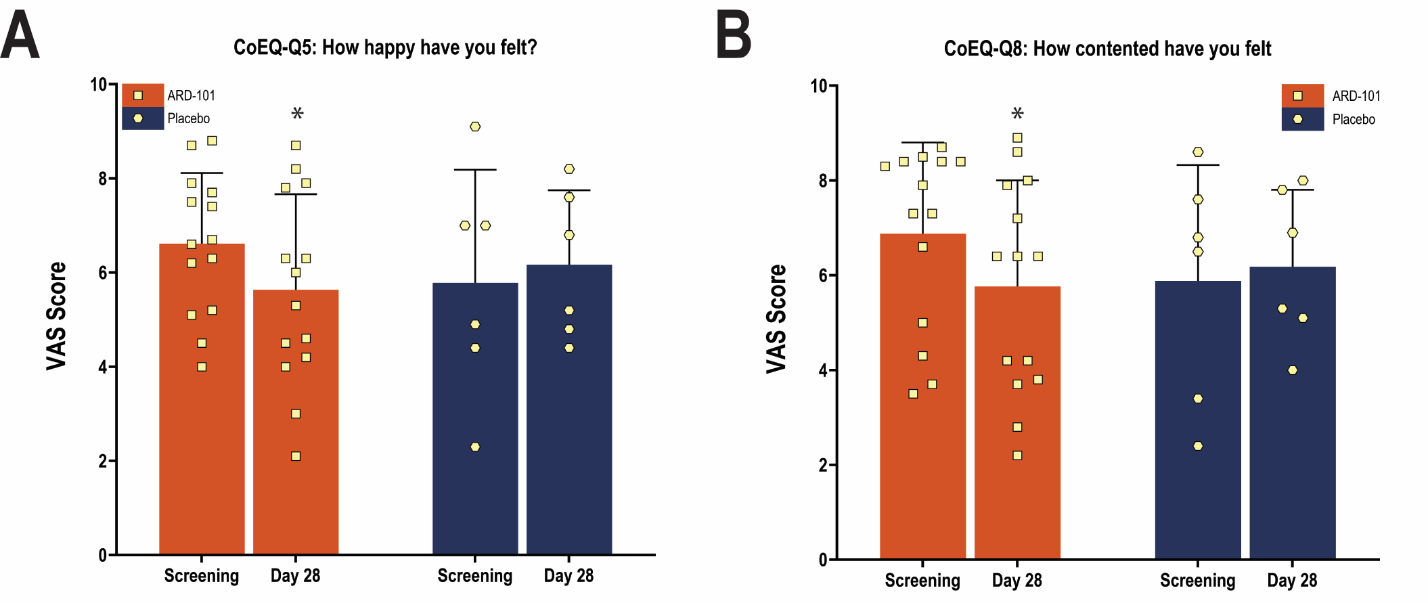


**Supplementary Figure 1. Minimal impact of ARD-101 on CoEQ items of eating-related positive mood in adults with obesity following ARD-101 treatment**. VAS scores for (A) Question 5 (happiness of eating) and (B) Question 8 (contentedness of eating) at Screening and Day 28. Data are shown as means (bars) and SEM (error bars) for ARD-101 (N = 14) and placebo (N = 6) groups, with individual values overlaid (dots). No significant main effect of treatment with ARD-101 vs. placebo on the change from baseline (Screening) to Day 28 was identified, while a significant within-subject variability was observed in the ARD-101 group (* *P* < 0.05).
